# A CRISPR-Cas13a–based two-step assay combined with lateral flow strips for rapid detection of Epstein–Barr virus

**DOI:** 10.3389/fmicb.2026.1850310

**Published:** 2026-05-22

**Authors:** Hui Liu, Xunqian Yin, Wenshan Duan, Haiqing Sun

**Affiliations:** 1 Clinical Laboratory Center of Beijing Youan Hospital, Capital Medical University, Beijing, China; 2 The College of Biomedical Sciences of Shandong First Medical University, Jinan, China; 3 The First Affiliated Hospital of China Medical University, Shenyang, China

**Keywords:** CRISPR/Cas13a, detection, EBV DNA, lateral flow strip, recombinase-aided amplification

## Abstract

**Background:**

Epstein–Barr virus (EBV) is a widespread oncogenic virus associated with multiple human malignancies. Accurate detection of EBV DNA is essential for disease screening and monitoring; however, commonly used methods such as quantitative PCR require specialized instruments and laboratory infrastructure, limiting their applicability in resource-limited settings.

**Methods:**

An isothermal nucleic acid detection method for EBV DNA was developed by combining recombinase-aided amplification with CRISPR/Cas13a detection. Target DNA was amplified under constant temperature conditions. Subsequently, Cas13a-mediated collateral RNase activity triggered the cleavage of labeled reporter molecules to produce detectable signals. Fluorescence measurement and lateral flow strip analysis were used for result interpretation.

**Results:**

The fluorescence assay detected EBV DNA across a dynamic range up to 10^7^ copies per reaction. The limit of detection (LoD) was established at 10^1^ copies per reaction. No cross-reactivity was observed. The lateral flow strip assay showed visible positive results at concentrations of 10^4^ copies per reaction and higher. In the preliminary feasibility evaluation using clinical blood samples, the fluorescence and lateral flow assays achieved positive detection rates of 100 and 84.6% (11/13), respectively, with both methods demonstrating no cross-reactivity against the tested control viruses.

**Conclusion:**

This study developed a two-step RAA–CRISPR/Cas13a assay for EBV DNA detection. The assay combines analytical sensitivity with an instrument-free visual readout. These findings demonstrate the technical feasibility of the RAA-CRISPR platform for EBV screening; however, further optimization is required to enhance the sensitivity of the visual readout. Large-scale clinical studies are essential to fully determine its diagnostic performance and practical utility in decentralized and resource-limited settings.

## 1 Introduction

EBV is a widespread human gammaherpesvirus that infects more than 95% of adults worldwide and establishes lifelong latent infection (Gao et al., 2025). Although primary EBV infection is usually mild or asymptomatic, persistent infection is strongly associated with several malignancies, including nasopharyngeal carcinoma, Hodgkin’s lymphoma, and gastric carcinoma (Zhang et al., 2009; MüNZ, 2025). EBV infection has also been linked to non-malignant diseases such as multiple sclerosis, highlighting its significant impact on global health (Bar-Or et al., 2020; Damania et al., 2022).

Because of its close association with disease development and progression, accurate detection of EBV DNA is essential for clinical diagnosis and disease monitoring. This is particularly important for nasopharyngeal carcinoma, which shows high incidence in certain geographic regions (Tan et al., 2020). At present, quantitative polymerase chain reaction (qPCR) is one of the most commonly used methods for EBV DNA detection. While this method provides high sensitivity and reliable quantification, it depends on expensive instruments, stable electricity, and trained personnel. These requirements limit its use in primary healthcare facilities and resource-limited settings, where accessible EBV testing is often most needed (Chinna et al., 2023). Therefore, there is a clear demand for simple, rapid, and portable methods for EBV DNA detection.

CRISPR-Cas systems offer rapid and isothermal molecular diagnostics. Specifically, the Cas13a enzyme is an RNA-guided RNase that provides highly specific signal amplification through target-triggered collateral RNA cleavage (Nouri et al., 2023; Yang et al., 2024). Because this assay detects EBV DNA, the double-stranded DNA amplicons generated by RAA are first converted into single-stranded RNA targets via T7 RNA polymerase-mediated transcription. Once guided by a specific crRNA to its target RNA, target-triggered Cas13a-mediated collateral RNase activity cleaves nearby reporter RNA molecules and generates a detectable signal. Cas13a-based assays can be combined with different signal readouts, including fluorescence and lateral flow strips, highlighting their potential for both laboratory testing and point-of-care applications (Hu et al., 2022).

To further simplify detection and avoid the need for thermal cycling, Cas13a detection is often combined with isothermal amplification methods. Recombinase-aided amplification (RAA) allows rapid nucleic acid amplification at a constant temperature and requires minimal equipment (Shang et al., 2024). By integrating RAA with CRISPR–Cas13a, target nucleic acids can be efficiently amplified and detected with high specificity (Tian et al., 2023). In addition, lateral flow strip–based readouts enable direct visual interpretation of results, further improving the practicality of this approach in decentralized testing environments.

In this study, we developed a two-step RAA–CRISPR/Cas13a assay for EBV DNA detection that incorporates both fluorescence-based and lateral flow strip–based readouts. We evaluated the analytical sensitivity and specificity of the assay using serially diluted EBV plasmids and control viral genomes. The preliminary feasibility of the method was further assessed using a small cohort of clinical blood samples from EBV-infected patients. As a proof-of-concept, this work aims to explore a flexible molecular diagnostic strategy for EBV detection. While further optimization and large-scale validation are required, the platform demonstrates potential for future applications in decentralized screening.

## 2 Materials and methods

### 2.1 Synthesis of plasmids, primers, and crRNAs

To ensure reliable detection of EBV, a total of 526 viral genome sequences were retrieved from the NCBI GenBank database, using the Epstein–Barr nuclear antigen 1 (*EBNA1*) gene as the primary target (GenBank accession no. NC_007605.1; Gene ID: 3783709). The retrieved sequences were aligned using SnapGene software. Based on the alignment, a conserved 272 bp region within the *EBNA1* gene, exhibiting >95% sequence conservation, was selected for assay development. This conserved region served as the template for designing the RAA primers and the corresponding CRISPR RNAs (crRNAs). To enable subsequent T7 transcription, a T7 promoter sequence was incorporated into the 5′ end of the forward RAA primer.

All primers and crRNAs were synthesized by Sangon Biotech (Shanghai, China) (Supplementary Figure 1). EBV plasmids containing the target sequence were synthesized based on the pUC57 vector (Biomed Biotechnology, China).

### 2.2 Recombinase-aided amplification

RAA reactions were performed using a commercial RAA kit (S001ZC, Hangzhou Zhongce Biotechnology, China) according to the manufacturer’s instructions. Reaction mixtures containing nuclease-free water, A buffer, and forward and reverse primers (10 μM each) were prepared and added to reaction tubes containing lyophilized RAA reagents. DNA templates were then added, followed by the addition of 2.5 μL B buffer to the tube caps.

After gentle mixing and brief centrifugation, the reaction tubes were incubated at 39 °C for 30 min in a constant-temperature heating block to allow isothermal amplification. After amplification, part of the products was purified using phenol–chloroform–isoamyl alcohol (25:24:1) extraction for agarose gel electrophoresis, while the remaining products were directly used for downstream CRISPR/Cas13a detection.

### 2.3 Agarose gel electrophoresis

RAA amplification products were analyzed on 2% agarose gels prepared in 1 × TAE buffer and stained with GelRed. Electrophoresis was performed at 100 V for 40 min. DNA bands were visualized using a Gel Doc XR + imaging system (Bio-Rad, USA) under ultraviolet illumination, and product sizes were verified using a DNA molecular weight marker.

### 2.4 CRISPR/Cas13a fluorescence assay

CRISPR/Cas13a fluorescence detection was carried out in a total reaction volume of 25 μL. Each reaction contained 80 nM LwaCas13a protein (GenScript, Z03486), 580 nM crRNA, 2 mM rNTP mix (New England Biolabs), 800 nM FAM-RNA-BHQ1 reporter probe, 1 × Cas13a reaction buffer, 10 mM HEPES, 20 mM MgCl₂, 1.5 U/μL T7 RNA polymerase (New England Biolabs), and 0.8 U/μL RNase inhibitor (New England Biolabs). Ten microliters of RAA amplification product was added as the input template, and nuclease-free water was used to adjust the final reaction volume (Supplementary Table 1). The reaction mixture was incubated at 37 °C for 60 min before fluorescence signal measurement.

### 2.5 CRISPR/Cas13a lateral flow strip assay

Lateral flow strip–based detection was performed using a CRISPR single-target nucleic acid test strip (31,203, TOLOBIO). According to the manufacturer’s specific design, the strip consists of a proximally located control line (C line) and a distally located test line (T line) along the flow direction. In the absence of Cas13a activation, intact reporter probes caused the gold nanoparticle complexes to be captured at the C line. Upon target-induced activation of Cas13a, the cleavage of reporter probes resulted in the appearance of a visible T line. The detection result was determined by the presence or absence of the T line.

The reaction procedure was performed with the following components: 80 nM LwaCas13a protein (GenScript, Z03486), 580 nM crRNA, 2 mM rNTP mix (New England Biolabs), 400 nM FAM-RNA-Biotin reporter probe, 1 × Cas13a reaction buffer, 10 mM HEPES, 20 mM MgCl₂, 1.5 U/μL T7 RNA polymerase (New England Biolabs), and 0.8 U/μL RNase inhibitor (New England Biolabs). Ten microliters of the RAA amplification product was added as the input template, and nuclease-free water was used to adjust the final reaction volume (Supplementary Table 2).

### 2.6 Analytical performance and clinical sample processing

Analytical sensitivity was evaluated using serially diluted EBV plasmids ranging from 1 to 10^7^ copies per reaction, analyzed by both fluorescence and lateral flow readouts. For the fluorescence assay, the positive cutoff threshold was defined as the mean fluorescence intensity of the no-template controls (NTCs) plus three times their standard deviation (mean + 3 SD). In this proof-of-concept study, the analytical LoD was empirically established as the lowest target concentration that yielded a fluorescence signal significantly above this cutoff threshold in all independent replicates.

To assess clinical feasibility and assay specificity, blood samples were collected from patients with confirmed EBV infection at Beijing Youan Hospital, Capital Medical University. Reference samples containing non-target viruses, including CMV, HSV-1, and VZV, were obtained from the same institution to evaluate potential cross-reactivity. This study was conducted in strict accordance with the principles of the Declaration of Helsinki. The protocol for the collection and use of all clinical specimens was reviewed and approved by the Ethics Committee of Beijing Youan Hospital (Approval No. LL-2025-057-K). Total viral DNA was extracted from the blood samples using the TIANamp Blood DNA Kit (TIANGEN Biotech, Beijing, China) strictly following the manufacturer’s protocol. The purified nucleic acids were aliquoted and stored at −80 °C. All clinical and non-target viral samples were subsequently tested under identical experimental conditions to systematically evaluate the assay’s preliminary clinical feasibility and cross-reactivity.

### 2.7 Contamination control

To minimize carry-over contamination, experiments were conducted in three physically separated areas: reagent preparation, RAA reaction setup, and CRISPR-based detection. A strict unidirectional workflow was maintained, with personnel and materials moving only from clean to post-amplification zones. Dedicated pipettes and aerosol-resistant filter tips were used throughout. Workspaces were routinely decontaminated with 75% ethanol and UV light. NTCs were included in every run to monitor for potential contamination.

### 2.8 Statistical analysis

The Cas13a-crRNA-fluorescence assay signal was expressed as the mean of ≥3 independent reactions ± SD. Statistical differences between the experimental groups and the NTC were evaluated using an unpaired two-tailed Student’s *t*-test. All statistical analyses and graphing were performed using GraphPad Prism software version 8.0 (Graph-Pad, Inc., La Jolla, CA, USA). A *p*-value of < 0.05 was considered to indicate statistical significance (**p < 0.01, ***p < 0.001, ****p < 0.0001).

## 3 Results

### 3.1 Optimization of the RAA-CRISPR detection assay

**Figure 1:**
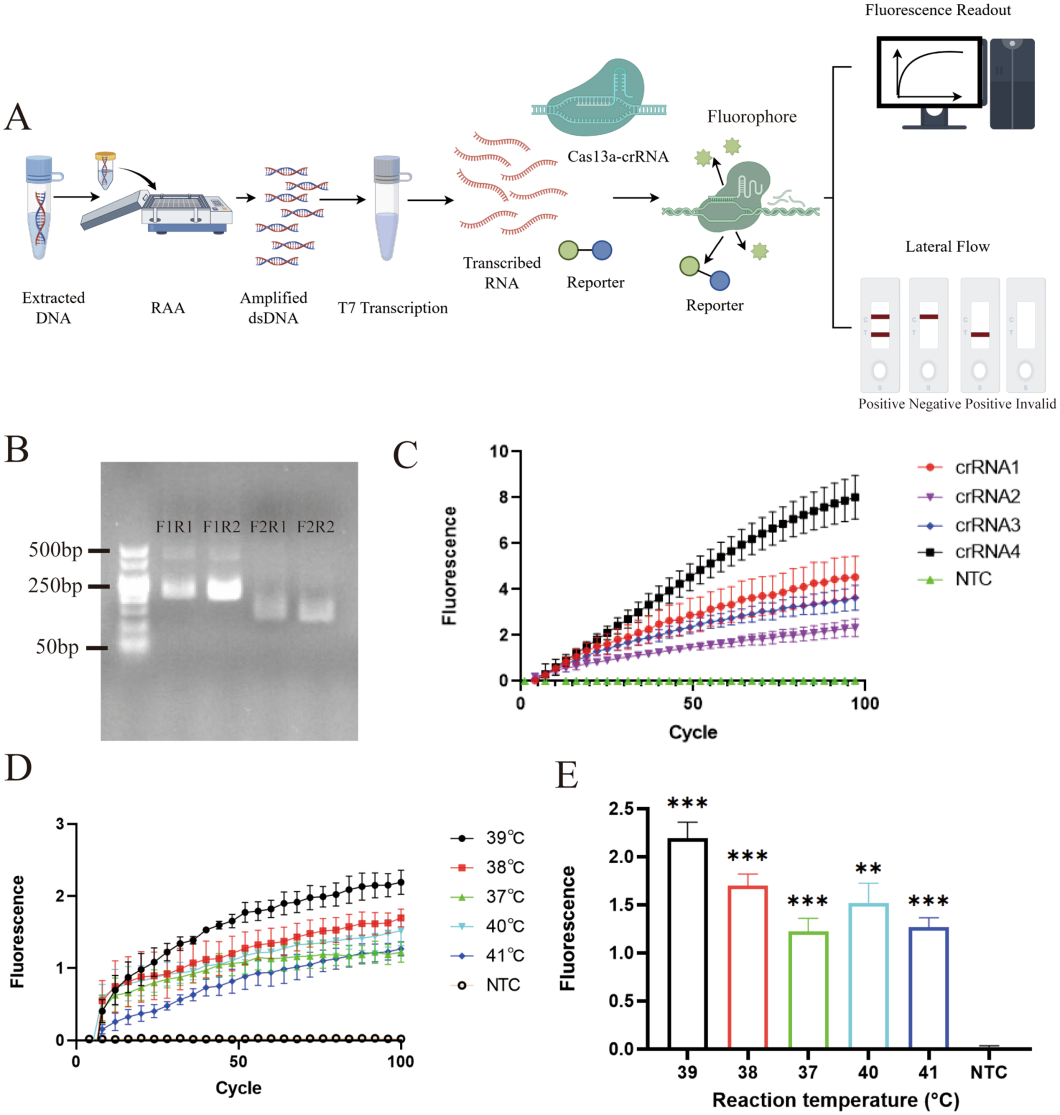
Development and optimization of the RAA–CRISPR/Cas13a detection platform. Data are representative of at least three independent experiments. Statistical significance was calculated relative to the NTC (**p < 0.01, ***p < 0.001). **(A)** Schematic overview of the experimental workflow and the trans-cleavage detection principle for EBV DNA. **(B)** Screening of RAA primer pairs using EBV-positive plasmid templates. **(C)** Selection of the optimal crRNA based on fluorescence signal intensity and cleavage kinetics (template concentration:10^9^ copies/L). **(D)** Effect of reaction temperature (37–41 °C) on RAA amplification efficiency, assessed by real-time and endpoint fluorescence signals(template concentration:10^7^ copies/L). **(E)** Endpoint fluorescence intensities comparing the assay performance across the tested temperature gradient.

The overall workflow of the RAA–CRISPR/Cas13a assay is illustrated in Figure 1A. To identify the optimal primer pair for EBV amplification, four RAA primer combinations (F1R1, F1R2, F2R1, and F2R2) were evaluated using EBV plasmid templates. Agarose gel electrophoresis showed that all primer pairs successfully amplified the target fragment. Among them, the F1R2 primer pair produced the strongest and most distinct amplification band, indicating higher amplification efficiency (Figure 1B). Therefore, F1R2 was selected for subsequent experiments.

Using the optimized RAA conditions, different crRNAs were further evaluated by fluorescence-based CRISPR/Cas13a detection. Among the tested crRNAs, crRNA4 generated the highest fluorescence signal and exhibited the most stable signal increase over time (Figure 1C). Based on these results, crRNA4 was selected as the optimal guide RNA for the detection system. To optimize the assay, RAA reactions were tested from 37 °C to 41 °C. While all tested temperatures produced positive fluorescent readouts, both the real-time cleavage kinetics (Figure 1D) and the endpoint fluorescence intensity analysis (Figure 1E) demonstrated that 39 °C achieved the most rapid signal accumulation and the highest maximal fluorescence output.

### 3.2 Analytical sensitivity of the RAA–CRISPR/Cas13a assay

**Figure 2:**
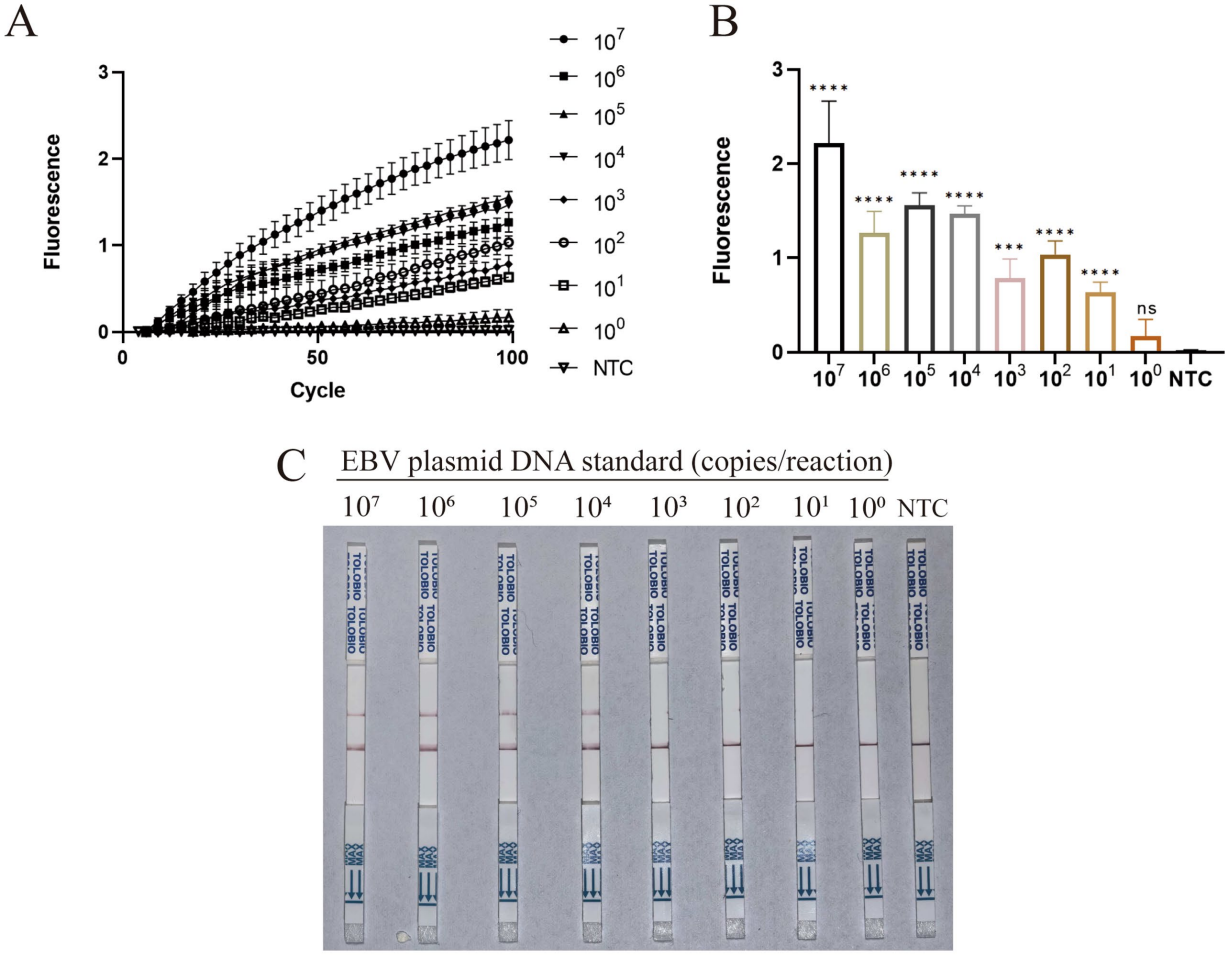
Analytical sensitivity of the two-step RAA–CRISPR/Cas13a assay. Data are representative of at least three independent experiments. Statistical significance was calculated relative to the NTC (***p < 0.001, ****p < 0.0001). **(A)** Real-time fluorescence kinetics evaluated using serially diluted EBV plasmid standards ranging from 10^7^ down to 10^0^ copies per reaction. **(B)** Endpoint fluorescence intensities corresponding to the serial dilutions, demonstrating the LoD of the fluorescence assay. **(C)** Visual evaluation of analytical sensitivity using the lateral flow strip assay with the identical serially diluted plasmid standards.

The analytical sensitivity of the two-step RAA–CRISPR/Cas13a assay was assessed using EBV plasmids serially diluted from 10^7^ down to 10^0^ copies per reaction. As shown in the real-time fluorescence kinetics (Figure 2A), the signal increased proportionally with the target template concentration. Based on the background fluorescence of the NTCs, the positive cutoff threshold was calculated to be 0.0841 (mean + 3SD). To precisely determine the analytical sensitivity, the endpoint fluorescence intensities (Figure 2B) were subjected to statistical analysis. The analysis established that the LoD of the fluorescence assay is 10^1^ copies per reaction, with the fluorescence signal remaining significant compared to the NTCs (****p < 0.0001).

Furthermore, the analytical sensitivity was visually evaluated using the lateral flow strip assay with the identical serially diluted plasmid standards. Clear and reproducible positive test lines were observed at target concentrations down to 10^4^ copies per reaction, whereas no T lines were detected for 10^3^ copies or the NTCs (Figure 2C). Therefore, the visual LoD of the lateral flow strip assay was also confirmed to be 10^4^ copies per reaction.

### 3.3 Analytical specificity of the RAA–CRISPR/Cas13a assay

**Figure 3:**
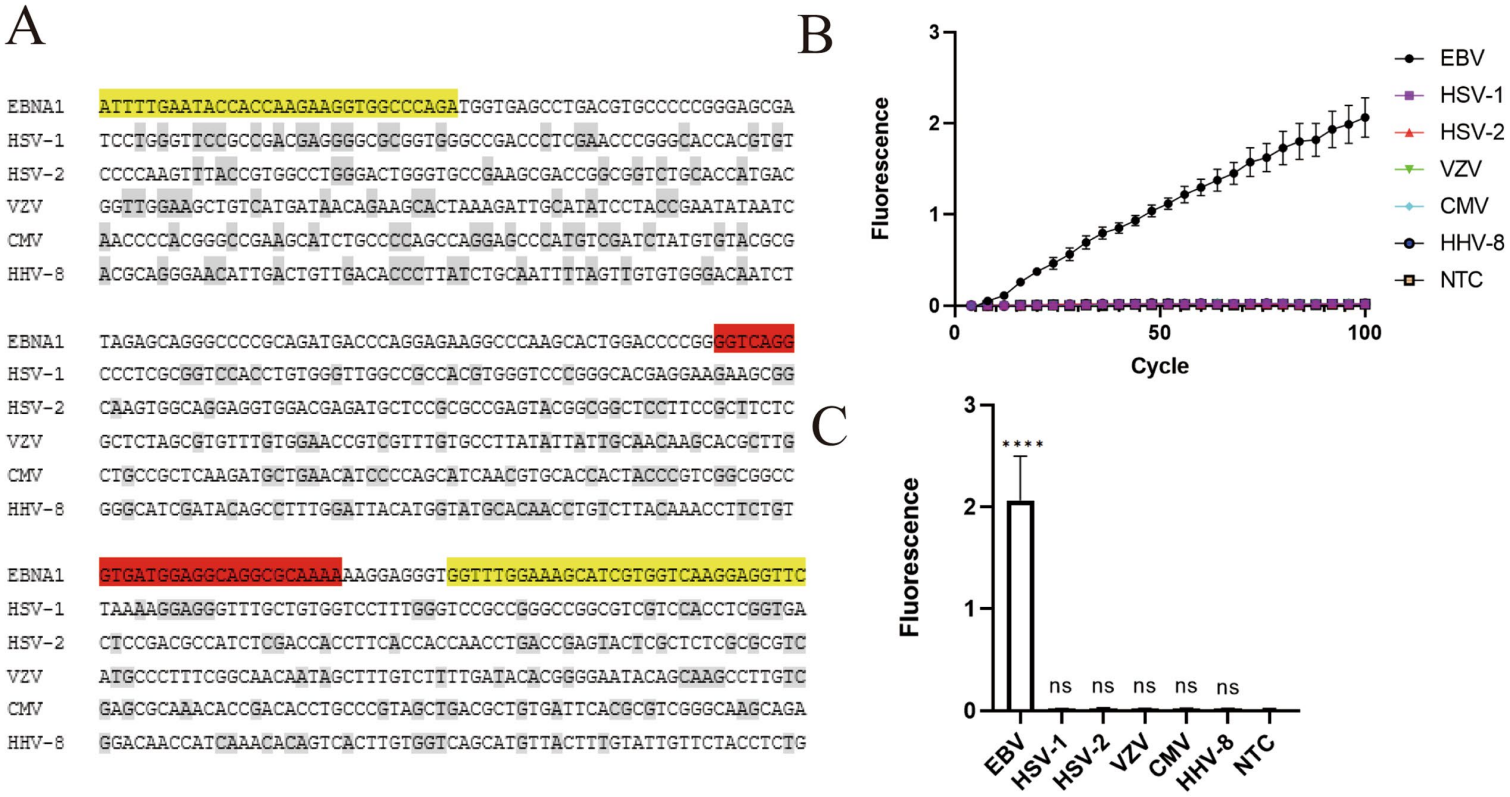
Analytical specificity of the two-step RAA–CRISPR/Cas13a assay. Data are representative of at least three independent experiments. Statistical significance was calculated relative to the NTC (****p < 0.0001). **(A)** The sequence alignment of the conserved *EBNA1* region of EBV with other common clinical herpesviruses (HSV-1, HSV-2, VZV, CMV, and HHV-8). The RAA primer and crRNA-binding regions are highlighted in yellow and red, respectively, showing no homologous sequences in non-target viruses. **(B)** Real-time fluorescence kinetics of the assay tested against EBV plasmid DNA and non-target viral targets. **(C)** Endpoint fluorescence signals of various viral targets.

To evaluate the analytical specificity of the assay, the sequence of the EBV *EBNA1* target region was first aligned with the corresponding genomic fragments of other common clinical herpesviruses, including HSV-1, HSV-2, VZV, CMV, and HHV-8. The RAA primer-binding regions and the crRNA-binding region are specific to EBV, exhibiting essentially no homologous sequences in the non-target viruses (Figure 3A).

The assay was evaluated using EBV plasmid DNA and the synthesized gene fragments of non-target viruses. The fluorescence signals for all synthesized non-target viral fragments remained at baseline, with no significant difference from the NTC (Figures 3B,C). These results demonstrate that the RAA–CRISPR/Cas13a assay does not cross-react with the synthesized fragments of other closely related herpesviruses.

### 3.4 Preliminary feasibility evaluation of CRISPR–Cas13a-based assay in clinical samples

**Figure 4:**
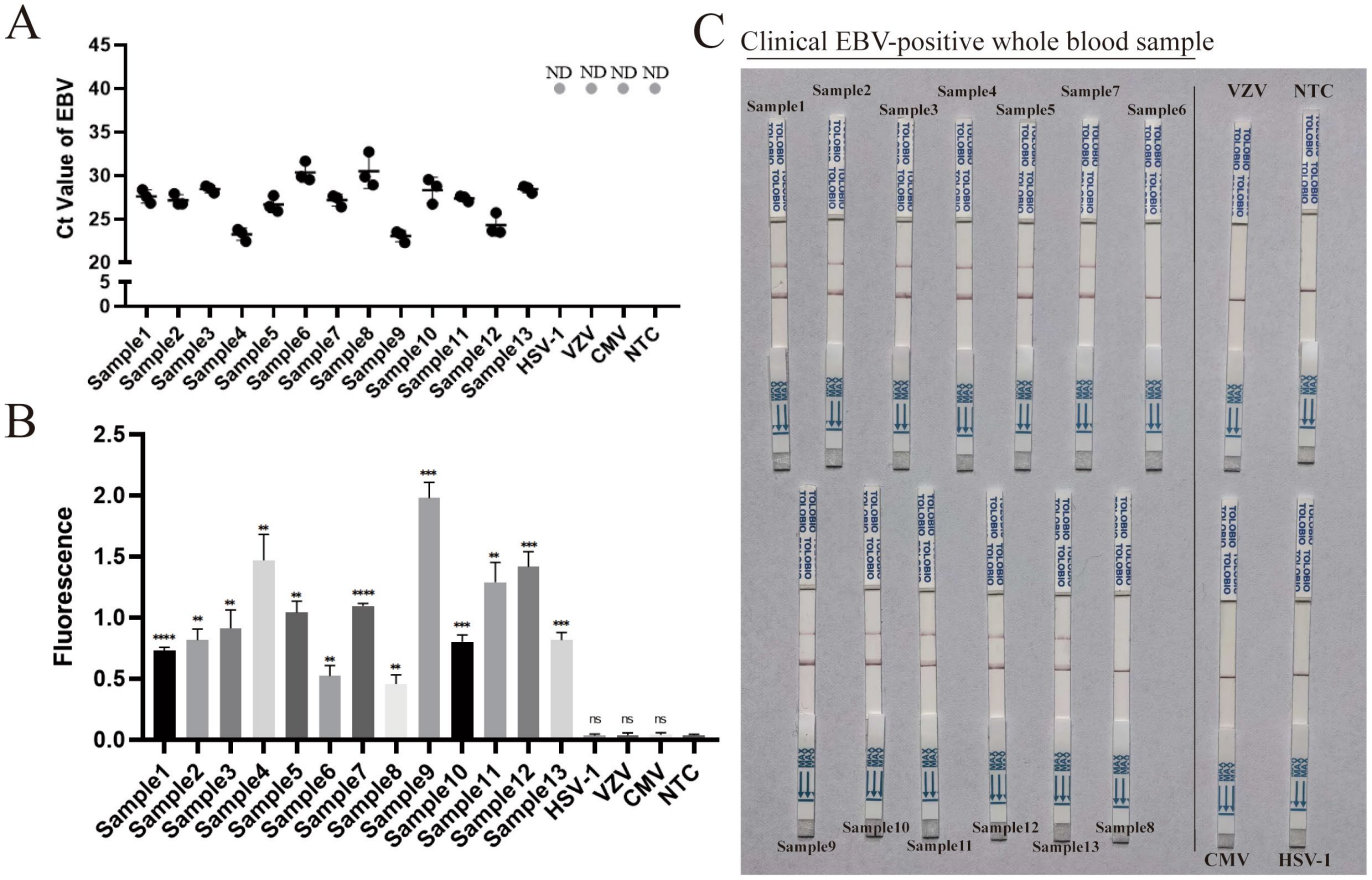
Preliminary feasibility evaluation using clinical samples for the two-step RAA–CRISPR/Cas13a assay. Data are representative of at least three independent experiments. Statistical significance was calculated relative to the NTC (**p < 0.01, ***p < 0.001, ****p < 0.0001). **(A)** Reference standard qPCR Ct values for 13 clinical EBV-positive whole blood samples and three non-target viral controls (HSV-1, VZV, and CMV). Undetected negative samples are denoted as ND. **(B)** Corresponding endpoint fluorescence intensities for the identical clinical cohort evaluated by the fluorescence assay. **(C)** Visual verification of the clinical samples using the lateral flow strip assay.

To evaluate the preliminary clinical feasibility of the assay, 13 EBV-positive whole blood samples and three non-target viral controls (HSV-1, VZV, and CMV) were tested. All 13 EBV samples tested positive by qPCR (Figure 4A), while the non-target controls and NTC were undetected (ND). Agarose gel electrophoresis confirmed successful RAA amplification in a clinical sample, with no amplification observed in the three non-target viral controls (Supplementary Figure 2).

These clinical samples were subsequently analyzed using the fluorescence assay. All 13 EBV-positive samples produced significantly higher fluorescence signals compared to the NTC (Figure 4B). The fluorescence values for the non-target viruses showed no significant difference from the NTC.

The samples were further evaluated using the lateral flow strip assay. Positive test lines appeared for 11 out of the 13 EBV-positive blood samples (Figure 4C). The three non-target viral samples and the NTC yielded negative results, with only the control line visible.

## 4 Discussion

In this study, we developed and analytically validated a two-step RAA–CRISPR/Cas13a platform for the detection of EBV DNA, incorporating both fluorescence-based and lateral flow strip–based readouts. By combining isothermal amplification with CRISPR/Cas13a-mediated sequence recognition, this platform enables sensitive and specific EBV detection while reducing dependence on complex laboratory instrumentation. These features establish a promising proof-of-concept for its future application in decentralized testing and point-of-care settings.

Accurate detection of EBV DNA is essential for the diagnosis and monitoring of EBV-associated diseases, particularly nasopharyngeal carcinoma and EBV-related lymphomas (Lin et al., 2004; Liu et al., 2025; Fung et al., 2016). Over the past few years, isothermal amplification coupled with CRISPR/Cas13a has been successfully applied in the molecular diagnosis of various viral pathogens, such as SARS-CoV-2, HIV, and HBV (Nouri et al., 2023; Patchsung et al., 2020; Zhu et al., 2025). While these pioneering studies have firmly established the analytical robustness of the Cas13a platform, its application for Epstein–Barr virus remains largely underexplored in point-of-care contexts. Compared to existing CRISPR-based viral assays, the technical novelty of our study lies in the rational design of crRNAs targeting a highly conserved 272-bp region of the *EBNA1* gene, combined with a flexible dual-readout system. Clinically, the current gold standards for EBV diagnosis rely heavily on serological testing, which suffers from delayed seroconversion windows, and qPCR, which is confined to centralized laboratories (Gao et al., 2025; Tan et al., 2020). By employing a CRISPR-based recognition mechanism, this assay provides a molecular screening method that functions independently of thermal cycling, offering potential clinical utility for decentralized EBV surveillance, particularly in resource-limited regions with a high incidence of EBV-associated malignancies (Wang et al., 2021; Plebani et al., 2025).

Isothermal amplification–based assays have attracted increasing attention for point-of-care testing because of their rapid reaction kinetics and minimal equipment requirements (Zhao et al., 2024). However, isothermal amplification alone may suffer from non-specific amplification, which can compromise result reliability. In this study, the introduction of the CRISPR/Cas13a system provided an additional layer of sequence-specific recognition following RAA amplification. Target-dependent activation of Cas13a collateral cleavage activity enhanced assay specificity and reduced the risk of false-positive results caused by non-specific amplification products (Yang et al., 2024).

The analytical performance of the proposed platform was systematically evaluated. Using the fluorescence-based readout, the assay achieved a limit of detection of 10^1^ copies per reaction. In the preliminary feasibility evaluation, this high sensitivity translated to a 100% detection rate (13/13) for EBV-positive blood samples. In parallel, the lateral flow strip readout enabled instrument-free visual detection with a limit of 104 copies per reaction. When applied to the same clinical cohort, the lateral flow assay successfully identified 11 out of 13 positive samples. The high clinical detection rate observed with the visual strips can be primarily attributed to the efficient recovery and concentration of viral targets from whole blood using the dedicated viral DNA extraction kit. This concentrated initial viral input, synergizing with the rapid exponential amplification during the initial RAA step, generated sufficient target amplicons to surpass the visual detection threshold. Specifically, as shown in Figure 4, the two EBV-positive samples that yielded negative results on the lateral flow strip corresponded to those with relatively higher qPCR Ct values. This observation is consistent with the explanation that the negative visual results were associated with lower initial target concentrations falling below the 10^4^ copies per reaction threshold. However, given the small sample size in this preliminary evaluation, this relationship should be interpreted with caution, and future studies with larger clinical cohorts are required to fully validate this correlation. Although the sensitivity of the lateral flow assay is lower than that of the fluorescence-based readout, it offers advantages in simplicity, portability, and ease of use, serving as a proof-of-concept with potential for future point-of-care applications in resource-limited settings where rapid visual readout is preferred (Patchsung et al., 2020).

Potential cross-reactivity was evaluated using nucleic acids from clinical viral controls (Li et al., 2023). No cross-reactivity was observed with these non-target viral samples in both fluorescence and lateral flow formats. This suggests a high degree of specificity provided by crRNA-guided Cas13a recognition for EBV detection under the tested conditions. Furthermore, while the assay showed no cross-reactivity with the selected viruses, its specificity against the entire human genome and a broader range of non-target pathogens has not been fully evaluated through extensive *in silico* analysis.

Several limitations of this study should be acknowledged. First, although the clinical feasibility was evaluated in a small patient cohort, large-scale clinical validation involving direct comparison with qPCR is required to fully evaluate its diagnostic performance and determine the correlation with viral load. Second, the sensitivity of the lateral flow strip assay remains lower than that of the fluorescence-based assay, and further optimization of reporter design or signal amplification strategies may help improve visual detection sensitivity (Wu et al., 2023). Furthermore, a critical limitation of the current assay design is its two-step format and the associated risk of carry-over contamination. The manual transfer of high-yield RAA amplicons between tubes renders the two-step assay susceptible to aerosolized DNA contamination. While spatial separation and unidirectional workflows prevented false positives in our laboratory evaluation, we acknowledge that the current design is primarily suitable for proof-of-concept testing. Practical deployment will strictly require closed-tube integration and the implementation of anti-contamination strategies. Recent pioneering studies have successfully demonstrated that optimizing buffer compatibility and enzyme kinetics can consolidate isothermal amplification and CRISPR cleavage into a single, closed reaction (Ding et al., 2020). Future research will focus on addressing these enzymatic incompatibilities to develop a fully integrated one-pot EBV diagnostic platform, thereby improving its biosafety and potential for point-of-care applications.

In summary, the current work establishes a preliminary RAA–CRISPR/Cas13a framework for EBV DNA detection. Our dual-readout system offers two distinct advantages depending on the testing environment. The fluorescence method ensures highly sensitive detection, whereas the lateral flow strip provides an equipment-free, visual alternative, albeit with a higher limit of detection. To translate this proof-of-concept into a reliable diagnostic tool for resource-limited settings, future efforts must focus on enhancing the visual detection sensitivity, integrating the two-step procedure into a closed-tube system to mitigate contamination risks, and conducting extensive validations in larger clinical cohorts. Despite these current limitations, this platform provides a foundational strategy that, with further technical refinement, holds potential for decentralized EBV screening.

## Data Availability

The original contributions presented in the study are included in the article/Supplementary material, further inquiries can be directed to the corresponding author.
