# Supplementary material for: A CRISPR-Cas13a–based two-step assay combined with lateral flow strips for rapid detection of Epstein–Barr virus
Source: Front Microbiol. 2026 May 22;17:1850310. doi: 10.3389/fmicb.2026.1850310 (PMC13236536; doi:10.3389/fmicb.2026.1850310)
Supplement: Supplementary file 1 [file Data_Sheet_1.pdf]

Table S1 RAA-CRISPR/Cas13a fluorescence detection System

Reagent	Concentration	Volume	Final Concentration
Cas13a	10 μM	0.2 μL	80 nM
crRNA	300 ng/μL	1 μL	12 ng/μL
rNTP Mix	25 mM	2 μL	2 mM
Cas13a reporter probe (FAM-RNA-BHQ1)	100 μM	0.2 μL	800 nM
Cas13a reaction buffer	10×	2.5 μL	1×
HEPES	1 M	0.25 μL	10 mM
T7 RNA Polymerase	50000 U/mL	0.75 μL	1.5 U/μL
RNase Inhibitor	40000 U/mL	0.5 μL	0.8 U/μL
$MgCl_2$	1 M	0.5 μL	20 mM
RAA product	—	10 μL	—
RNase-free water	—	Up to 25 μL	—

Table S1. Components and optimized reaction system for the RAA-CRISPR/Cas13a fluorescence assay. This table details the specific reagents, initial concentrations, added volumes, and final concentrations required for the 25 μL fluorescence detection system.

Table S2 RAA-CRISPR/Cas13a lateral flow strip detection system

Reagent	Concentration	Volume	Final Concentration
Cas13a	10 μM	0.4 μL	80 nM
crRNA	300 ng/μL	2 μL	12 ng/μL
rNTP Mix	25 mM	4 μL	2 mM
Cas13a strip probe (FAM-RNA-Biotin)	100 μM	0.2 μL	400 nM
Cas13a reaction buffer	10×	5 μL	1×
HEPES	1 M	0.5 μL	10 mM
T7 RNA Polymerase	50000 U/mL	1.5 μL	1.5 U/μL
RNase Inhibitor	40000 U/mL	1 μL	0.8 U/μL
$MgCl_2$	1 M	1 μL	20 mM
RAA product	—	10 μL	—
RNase-free water	—	Up to 50 μL	—

Table S2. Components and optimized reaction system for the RAA-CRISPR/Cas13a lateral flow dipstick assay. This table details the specific reagents, initial concentrations, added volumes, and final concentrations required for the 50 μL visual detection system using lateral flow strips.

F1 R1 crRNA1
GCGCAGGCCCCCTCCAGGTAGAAGGCCATTTTTCCACCCTGTAGGGGAAGCCGATTATTTTGAA
TACCACCAAGAAGGTGGCCCAGATGGTGAGCCTGACGTGCCCCCGGGAGCGATAGAGCAGGG
CCCCGCAGATGACCCAGGAGAAGGCCCAAGCACTGGACCCCGGGGTCAGGGTGATGGAGGCA
GGCGCAAAAAAGGAGGGTGGTTTGGAAAGCATCGTGGTCAAGGAGGTTCCAACCCGAAATTTG
AGAACATTGCAGAAGGTTTAAGA

F2 R2 crRNA2
GCGCAGGCCCCCTCCAGGTAGAAGGCCATTTTTCCACCCTGTAGGGGAAGCCGATTATTTTGAA
TACCACCAAGAAGGTGGCCCAGATGGTGAGCCTGACGTGCCCCCGGGAGCGATAGAGCAGGG
CCCCGCAGATGACCCAGGAGAAGGCCCAAGCACTGGACCCCGGGGTCAGGGTGATGGAGGCA
GGCGCAAAAAAGGAGGGTGGTTTGGAAAGCATCGTGGTCAAGGAGGTTCCAACCCGAAATTTG
AGAACATTGCAGAAGGTTTAAGA

crRNA3
GCGCAGGCCCCCTCCAGGTAGAAGGCCATTTTTCCACCCTGTAGGGGAAGCCGATTATTTTGAA
TACCACCAAGAAGGTGGCCCAGATGGTGAGCCTGACGTGCCCCCGGGAGCGATAGAGCAGGG
CCCCGCAGATGACCCAGGAGAAGGCCCAAGCACTGGACCCCGGGGTCAGGGTGATGGAGGCA
GGCGCAAAAAAGGAGGGTGGTTTGGAAAGCATCGTGGTCAAGGAGGTTCCAACCCGAAATTTG
AGAACATTGCAGAAGGTTTAAGA

crRNA4
GCGCAGGCCCCCTCCAGGTAGAAGGCCATTTTTCCACCCTGTAGGGGAAGCCGATTATTTTGAA
TACCACCAAGAAGGTGGCCCAGATGGTGAGCCTGACGTGCCCCCGGGAGCGATAGAGCAGGG
CCCCGCAGATGACCCAGGAGAAGGCCCAAGCACTGGACCCCGGGGTCAGGGTGATGGAGGCA
GGCGCAAAAAAGGAGGGTGGTTTGGAAAGCATCGTGGTCAAGGAGGTTCCAACCCGAAATTTG
AGAACATTGCAGAAGGTTTAAGA

F1 taatacgactcactatagggATTTTGAATACCACCAAGAAGGTGGCCCAGA
R1 GGTTGGAACCTCCTTGACCACGATGCTTTCC
F2 taatacgactcactatagggGATGACCCAGGAGAAGGCCCAAGCACTGGAC
R2 GAACCTCCTTGACCACGATGCTTTCCAAACC

CrRNA1
GAUUUAGACUACCCCAAAAACGAAGGGGACUAAAACUCCUUUUUUGCGCCUGCCUCCAUCA
CCC

CrRNA2
GAUUUAGACUACCCCAAAAACGAAGGGGACUAAAACUUUUGCGCCUGCCUCCAUCACCCUG
ACC

CrRNA3
GAUUUAGACUACCCCAAAAACGAAGGGGACUAAAACCGGGGUCCAGUGCUUGGGCCUUCU
CCUG

CrRNA4
GAUUUAGACUACCCCAAAAACGAAGGGGACUAAAACUGCGCCUGCCUCCAUCACCCUGACC
CCG

Figure S1. Detailed sequence designs for RAA primers and Cas13a crRNAs targeting the EBV *EBNA1* gene. The comprehensive sequence information of the target regions, RAA forward and reverse primers (F1/R1, F2/R2), and Cas13a crRNAs (crRNA1–4) is provided.

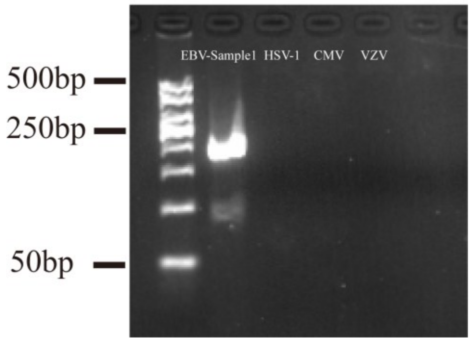

Figure S2. Specificity verification of the RAA amplification stage via agarose gel electrophoresis. To confirm that the high specificity of the assay originates from the initial amplification step, RAA products from an EBV-positive clinical sample (Sample 1) and non-target clinical viral samples (HSV-1, CMV, VZV) were analyzed. A distinct target amplicon band is exclusively observed in the EBV-positive sample lane. No detectable cross-reactivity was observed with the selected herpesvirus controls under the tested conditions during the isothermal amplification process.
